# Maintaining ideal cardiovascular health is associated with higher serum anti-aging protein klotho in the middle-aged and older populations

**DOI:** 10.1016/j.jnha.2024.100224

**Published:** 2024-04-05

**Authors:** Kaisaierjiang Kadier, Pengfei Liu, Diliyaer Dilixiati, Xinliang Peng, Aikeliyaer Ainiwaer, Dinigeer Kadier, Jiande Lu, Xiaozhu Liu, Mierxiati Ainiwan, Qi Wang, Xiang Ma, Yitong Ma

**Affiliations:** a Department of Cardiology, First Affiliated Hospital of Xinjiang Medical University, Urumqi, China; b Department of Urology, First Affiliated Hospital of Xinjiang Medical University, Urumqi, China; c Department of Physiology, Cardiovascular Research Institute Maastricht (CARIM), Maastricht, Netherlands; d Data Science and Business Analytics, Faculty of Economics and Business, University of Amsterdam, Amsterdam, Netherlands; e Department of General Surgery, Children's Hospital of Xinjiang Uygur Autonomous Region, Urumqi, China; f Department of Critical Care Medicine, Beijing Shijitan Hospital, Capital Medical University, Beijing, China

**Keywords:** Cardiovascular health, Life’s Essential 8, Klotho, Biomarker of aging, NHANES

## Abstract

**Objectives:**

Maintaining ideal cardiovascular health (CVH) is believed to have potential anti-aging benefits. The American Heart Association (AHA) recently updated the “Life's Essential 8 (LE8)” metrics to measure ideal CVH, but its connection with the anti-aging protein klotho is still unclear. We aimed to explore the relationship between ideal cardiovascular health and serum anti-aging protein klotho in a nationally representative US middle-aged and older population.

**Design:**

A cross-sectional study.

**Setting:**

The National Health and Nutrition Examination Survey (2007−2016).

**Participants:**

A total of 9457 middle-aged and older participants.

**Measurements:**

Ideal CVH scores and their components were defined according to the guidelines set by the AHA. Serum klotho detected by enzyme-linked immunosorbent assay. Weighted multivariable linear regression and restricted cubic spline were employed to examine the association between CVH score and klotho. Subgroup analyses were conducted, stratified by age (40−59 and 60−79), sex (Male and Female), race (Mexican American, non-Hispanic White, non-Hispanic Black, and Others) and chronic kidney disease (Yes and No) in fully adjusted models.

**Results:**

A total of 9457 middle-aged and older participants were included in this study, with a mean age of 55.27 ± 0.17 years. The mean serum klotho level in the population was 849.33 ± 5.39 pg/mL. After controlling for potential confounders, the LE8 score showed a positive correlation with serum klotho levels (β: 1.32; 95% CI 0.73, 1.91), and a non-linear dose-response relationship was observed. Furthermore, we also discovered a positive relationship between health behaviors score and health factors score and serum klotho levels (β: 0.48; 95% CI 0.07, 0.88 and β: 1.05; 95% CI 0.54, 1.56, respectively), particularly a stronger correlation between health factors and serum klotho. In the subgroup analysis, we observed a significant interaction between LE8 score and sex and race. (P for interaction <0.05).

**Conclusions:**

LE8 and its subscale scores were positively associated with serum klotho levels in the middle-aged and older populations. Promoting the maintenance of ideal CVH can contribute to delaying the aging process.

## 1 Introduction

At present, aging speeds up the development and advancement of age-related diseases, especially cardiovascular disease (CVD) [1]. Aging is an unavoidable process, but it has been confirmed that maintaining optimal cardiovascular health (CVH) is crucial in preventing and slowing down the process of biological aging [1,2].

Recently, the AHA has updated algorithms to assess CVH and introduced sleep as a new component in the proposed “Life’s Essential 8 (LE8)”[3]. Subsequent studies have consistently shown that maintaining a high CVH score is strongly associated with increased life expectancy and can effectively slow down the aging process [2,4]. Aging is the gradual deterioration of an organism's overall condition, characterized by molecular and cellular changes in the body, along with the emergence of aging biomarkers [5]. Klotho, which regulates oxidative stress and aging-related signaling pathways in vivo, is currently a prominent focus of aging marker research [6]. Multiple studies have discovered a significant correlation between lower levels of circulating klotho and increased mortality rates [7,8]. In addition, klotho is closely associated with cardiovascular risk factors such as high BMI, smoking, alcohol consumption, and lipid parameters [9]. However, to date, no study has been able to establish a connection between maintaining optimal CVH and the levels of serum klotho. Therefore, we conducted a study to examine the relationship between LE8 score, a measure of CVH, and serum klotho levels in a representative sample of middle-aged and older in the United States.

## 2 Materials and methods

### 2.1 Study population

Data for this study were obtained from the National Health and Nutrition Examination Survey (NHANES) database, which covers the period from 2007 to 2016. NHANES is a national survey conducted in the United States to gather information about the noninstitutionalized civilian population. The survey uses a complex, stratified, and multistage probability sampling design to ensure representative results [10]. To learn more about the NHANES survey and access detailed information, please visit the official website of the Centers for Disease Control and Prevention at https://www.cdc.gov/nchs/index.htm. The NHANES study protocol has received approval from the National Center for Health Statistics Research Ethics Review Board, and all participants have given written informed consent. The study was conducted in accordance with the Strengthening the Reporting of Observational Studies in Epidemiology guidelines, which provide a framework for reporting cross-sectional studies [11].

**Fig. 1:**
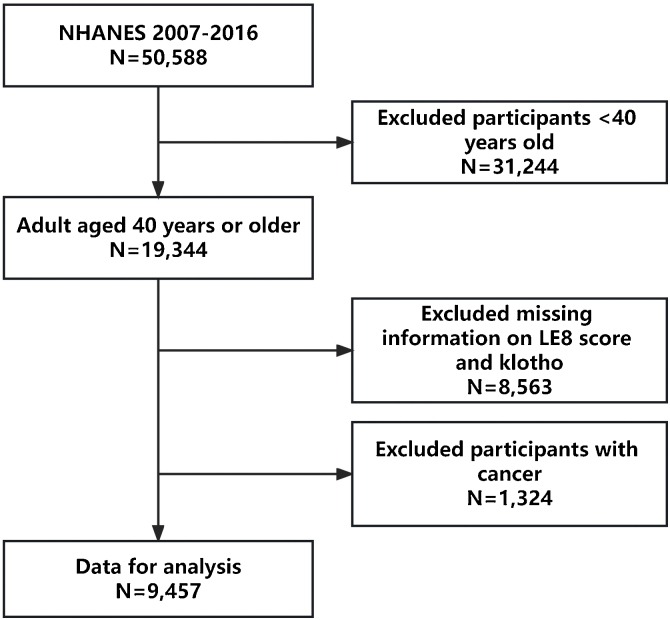
Flow chart of eligible National Health and Nutrition Examination Survey (NHANES) participants included in this study. LE8, Life’s Essential 8.

The survey was limited to individuals in the United States who were middle-aged and older, specifically those aged 40–79 years. Serum klotho levels were not measured in participants aged <40 years. After excluding participants who were aged <40 years (n = 31,244), participants with missing serum klotho data (n = 5580), participants with missing data on ideal CVH indicators (n = 2983), and participants who self-reported having cancer (n = 1324), a total of 9457 participants were included in the final analysis **(**Fig. 1). **Supplementary Table 1** outlines the characteristics of participants who were excluded from this study due to missing data.

### 2.2 Definitions of LE8 metrics

The AHA has recently updated the concept of ideal CVH metrics, which is now referred to as LE8 [3]. LE8 metrics encompasses four health behaviors (balanced diet, regular physical activity, avoiding tobacco exposure and ideal sleep duration) and four health factors (Body mass index (BMI), blood pressure, blood glucose, and non-high-density lipoprotein cholesterol). For a detailed explanation of how each index score in LE8 is calculated using the NHANES database, please refer to **Supplementary Table 2** [12]. Dietary measures were evaluated using the 2015 Healthy Eating Index (HEI-2015), which was assessed through a 24-h dietary recall of participants [13]. Information on physical activity, nicotine exposure, sleep patterns, history of diabetes, and medication usage was collected through self-report questionnaires. Height, weight, and blood pressure were measured during examinations. BMI was calculated as weight (kilogram) divided by height (metre) squared. Blood samples were collected to measure non-high-density lipoprotein cholesterol, glucose, and glycated hemoglobin levels. Each measure in the LE8 score ranges from 0 to 100 points, and the total LE8 score is calculated by taking the unweighted average of the eight measures. Participants were categorized as having high CVH if their total LE8 score was between 80 and 100, moderate CVH if their score was between 50 and 79, and low CVH if their score was between 0 and 49 [12]. At the same time, health behaviors, health factors, and the remaining 8 subscales were classified using the same principles. LE8 and its submetrics were considered as exposure variables in this study.

### 2.3 Measurement of serum klotho concentrations

The NHANES 2007–2016 cycle collected frozen serum samples primarily for Soluble alpha-klotho analysis in individuals aged 40 years and above, which were stored at temperatures below −80 °C. These serum samples were received and tested at the Northwestern Lipid Metabolism and Diabetes Research Laboratory at the University of Washington, Seattle, between 2019 and 2020. Klotho levels were measured using an ELISA kit (IBL International, Gunma, Japan) [14]. Specific laboratory techniques and quality control procedures are detailed in the NHANES Laboratory Procedures Manual. Serum klotho was regarded as an outcome variable in this study.

### 2.4 Covariates

As per the existing literature [15,16], the study encompassed the following covariates: age, sex, race, education, marital status, poverty-income ratio (PIR), insurance status, alcohol consumption status, family history of cardiovascular disease (HCVD), and chronic kidney disease (CKD). In-home interviews were conducted by trained NHANES interviewers using a computer-assisted system to collect demographic information. Demographic covariates in this study included age (40−59 and 60−79), sex (Male and Female), race (Mexican American, non-Hispanic White, non-Hispanic Black, and Others), marital status (Married/Living with Partner, Widowed/Divorced/ Separated and Never married), education (Less than high school, High school and Above high school), PIR (<1.3, 1.3−3.5 and >3.5), and insurance coverage status (Yes and No). Alcohol consumption status (Never, Former, Mild-Moderate and Heavy) and family history of cardiovascular disease (HCVD) (Yes and No) were obtained through self-reported questionnaires. According to the International Renal Association, CKD is characterized by a glomerular filtration rate of less than 60 mL/min/1.73 m^2^ or a urine albumin-creatinine ratio of at least 30 [17].

### 2.5 Statistical analysis

All statistical analyses conducted in this study took into account the complex multistage probability sampling design of NHANES. This included incorporating survey design variables and sampling weights to appropriately account for the study design mentioned above [10]. We refined the analysis by adjusting the weights of mobile examination centers. This was done by dividing the weights by five, as the data covered five survey cycles and ten survey years.

Descriptive statistical analyses were conducted to present the characteristics of participants based on the classification of ideal CVH. The weighted mean ± standard deviation (SD) was used to present continuous variables and compared using one-way ANOVA. Categorical variables were compared using the Rao-Scott chi-square test and presented as weighted percentage (95% confidence interval, 95% CI). Linear regression was employed to assess the relationship between klotho as the dependent variable and LE8 score and its subscales as independent variables. Beta coefficients and 95% CIs were computed. Further analyses involved incorporating three categories of high CVH, moderate CVH, and low CVH for the LE8 score and its subscales into linear regression models. Categorical variables were included in the analysis of the model, with low CVH as the reference. The medians of the three groups were also included in the model as continuous variables for trend testing. Mode 1 is the unadjusted model. Model 2 was adjusted for age, sex, and race. Model 3 was adjusted for the parameters in Model 2 plus education, marital status, PIR, insurance status, alcohol consumption status, HCVD, and CKD. We utilized the vif() function in R software to assess the variance inflation factor for each covariate. The results indicated that all variables had a variance inflation factor of less than 5, indicating the absence of multicollinearity among the covariates.

Subgroup analyses were conducted, stratified by age, sex, race and CKD in fully adjusted models. Additionally, likelihood ratio tests were used to assess multiplicative interactions. In the fully adjusted model, we employed the restricted cubic spline (RCS) method with four nodes positioned at the 5th, 35th, 65th and 95th percentiles to investigate the non-linear association between the LE8 score, serving as the exposure variable, and the klotho outcome variable.

Missing values for covariates, whether continuous or categorical, were imputed in this study using the MissForest package, a widely-used imputation method in statistical analysis [18]. **Supplementary Table 3** provides comprehensive data on the missing covariates. Sensitivity analyses were conducted with the following criteria: (1) only participants with complete covariate data were included; (2) participants with CVD were excluded. Statistical analysis was conducted using R software (version 4.1.3; https://www.R-Project.org), incorporating complex sampling modules. Results were deemed statistically significant when the P-values from two-sided tests were below 0.05 for all statistical analyses.

## 3 Results

### 3.1 Baseline characteristics

This study involved 9457 participants aged between 40 and 79 years, representing 79,159,178 noninstitutionalized residents in the United States within this age group. Overall, participants had a mean age ± SD of 55.27 ± 0.17 years, with 52.95% (95% CI 48.82–57.09) being female and 73.79% (95% CI 65.53–82.04) identifying as non-Hispanic white. Table 1displays the baseline characteristics of participants categorized by CVH. Serum klotho levels exhibited a mean value of 849.33 ± 5.39 pg/mL in the general population. Notably, these levels varied across the low, moderate, and high CVH groups, indicating a positive correlation between higher CVH levels and elevated serum klotho levels. Participants with low and moderate CVH were more likely to be older, male, have a lower educational level, lower PIR, and lower insurance coverage compared to those with high CVH. Additionally, there were differences in race, marital status, alcohol consumption status, HCVD and CKD between the CVH groups (all P < 0.05).

**Table 1:** General characteristics of the included participants (n = 9457) in the NHANES 2007–2016.

Characters	Overall (n = 9457)	Low LE8 score (0–49) (n = 1580)	Moderate LE8 score (50–79) (n = 6437)	High LE8 score (80–100) (n = 1440)	P-value
Klotho(pg/mL)	849.33 ± 5.39	818.97 ± 9.81	842.82 ± 6.37	891.41 ± 11.46	< 0.0001
Age,year	55.27 ± 0.17	56.96 ± 0.30	55.52 ± 0.20	53.30 ± 0.42	< 0.0001
Age,year					< 0.0001
60−79	33.64(30.58−36.69)	40.14(36.53−43.75)	34.39(32.59−36.18)	26.79(22.95−30.63)	
40−59	66.36(61.08−71.65)	59.86(56.25−63.47)	65.61(63.82−67.41)	73.21(69.37−77.05)	
Sex					< 0.0001
Male	47.05(43.08−51.01)	45.74(41.94−49.55)	49.98(48.50−51.47)	38.03(34.91−41.15)	
Female	52.95(48.82−57.09)	54.26(50.45−58.06)	50.02(48.53−51.50)	61.97(58.85−65.09)	
Race					< 0.0001
Mexican American	6.53(5.26−7.80)	6.65(4.41−8.90)	7.15(5.57−8.73)	4.36(3.36−5.36)	
Non-Hispanic Black	9.39(8.26−10.53)	14.98(11.98−17.99)	9.79(8.21−11.38)	4.32(3.36−5.28)	
Non-Hispanic White	73.79(65.53−82.04)	68.65(63.93−73.37)	72.85(69.51−76.19)	80.35(77.64−83.07)	
others	10.29(9.14−11.44)	9.72(7.34−12.09)	10.20(8.70−11.70)	10.97(9.05−12.90)	
Marital					< 0.0001
Married/Living with Partner	71.53(65.21−77.86)	62.77(58.95−66.59)	70.42(68.84−72.00)	81.11(78.83−83.39)	
Widowed/Divorced/ Separated	21.04(19.32−22.77)	27.24(24.25−30.24)	22.08(20.73−23.42)	13.44(11.50−15.39)	
Never married	7.42(6.53−8.32)	9.98(7.17−12.80)	7.50(6.48−8.53)	5.44(3.99−6.89)	
Education					< 0.0001
Less than high school	15.72(13.93−17.51)	28.29(24.77−31.81)	15.92(14.03−17.81)	6.70(5.14−8.26)	
High school	22.80(20.23−25.37)	31.52(27.16−35.87)	24.56(22.74−26.38)	11.08(8.92−13.25)	
Above high school	61.48(55.88−67.08)	40.20(35.75−44.64)	59.52(56.64−62.40)	82.21(79.38−85.05)	
Poverty-income ratio					< 0.0001
< 1.3	15.43(13.84−17.01)	29.41(26.01−32.80)	15.40(13.59−17.21)	6.24(4.77−7.72)	
1.3−3.5	36.81(33.58−40.04)	42.52(39.10−45.93)	38.31(36.01−40.61)	27.98(24.35−31.60)	
> 3.5	47.76(42.36−53.16)	28.08(23.59−32.56)	46.29(43.21−49.37)	65.78(61.63−69.93)	
Insurance					< 0.0001
No	13.25(12.00−14.49)	16.09(13.89−18.29)	13.90(12.54−15.27)	9.14(7.28−11.01)	
Yes	86.75(79.56−93.94)	83.91(81.71−86.11)	86.10(84.73−87.46)	90.86(88.99−92.72)	
Alcohol consumption status					< 0.0001
Never	10.38(9.19−11.57)	9.26(7.61−10.90)	10.62(9.46−11.79)	10.32(8.16−12.48)	
Former	17.49(15.83−19.14)	30.93(27.57−34.29)	17.19(15.85−18.54)	9.54(7.54−11.54)	
Mild-Moderate	39.69(36.03−43.35)	28.88(25.09−32.67)	38.46(36.50−40.42)	51.00(46.94−55.07)	
Heavy	32.44(29.33−35.56)	30.93(27.59−34.27)	33.73(32.02−35.43)	29.13(25.51−32.75)	
HCVD					< 0.0001
No	85.37(78.74−92.01)	77.00(73.94−80.05)	85.96(84.75−87.17)	88.97(87.22−90.72)	
Yes	14.63(13.07−16.18)	23.00(19.95−26.06)	14.04(12.83−15.25)	11.03(9.28−12.78)	
CKD					< 0.0001
No	85.42(78.44−92.39)	71.91(69.05−74.76)	86.06(84.86−87.25)	92.24(90.28−94.21)	
Yes	14.58(13.26−15.90)	28.09(25.24−30.95)	13.94(12.75−15.14)	7.76(5.79−9.72)	
Health behaviors score	67.32 ± 0.45	39.00 ± 0.57	67.00 ± 0.33	87.22 ± 0.32	< 0.0001
Health factors score	65.84 ± 0.33	44.74 ± 0.44	64.12 ± 0.28	85.67 ± 0.35	< 0.0001
HEI-2015 diet score	42.72 ± 0.62	20.94 ± 0.86	39.56 ± 0.59	67.82 ± 0.98	< 0.0001
Physical activity score	70.60 ± 0.78	24.78 ± 1.54	72.16 ± 0.78	95.76 ± 0.39	< 0.0001
Nicotine exposure score	72.33 ± 0.63	43.83 ± 1.31	72.38 ± 0.69	91.10 ± 0.72	< 0.0001
Sleep health score	83.65 ± 0.37	66.46 ± 1.02	83.91 ± 0.33	94.20 ± 0.38	< 0.0001
Body mass index score	58.02 ± 0.55	32.43 ± 1.06	55.67 ± 0.57	82.96 ± 0.70	< 0.0001
Blood lipids score	58.71 ± 0.43	42.75 ± 1.13	56.50 ± 0.48	76.77 ± 0.87	< 0.0001
Blood glucose score	81.96 ± 0.43	58.37 ± 0.87	82.32 ± 0.42	96.44 ± 0.42	< 0.0001
Blood pressure score	64.67 ± 0.51	45.40 ± 1.04	61.99 ± 0.57	86.52 ± 0.72	< 0.0001

Abbreviations: LE8, Life’s Essential 8; HCVD, Family history of cardiovascular disease; CKD, Chronic kidney disease; HEI-2015, 2015 Healthy Eating Index.

Continuous variables are presented as the weighted mean ± standard deviation, and were compared using the weighted one-way ANOVA test.

Categorical variables are presented as weighted percentages (95% confidence interval), and were compared using the weighted Rao-Scott chi-square test.

### 3.2 Association between LE8 score and serum klotho

**Fig. 2:**
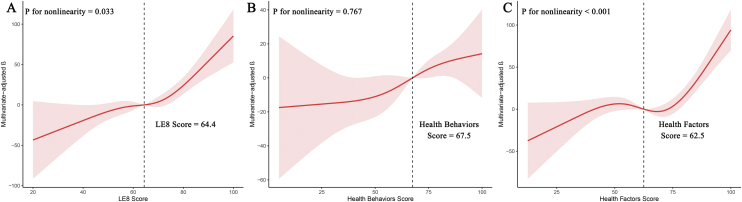
Nonlinear relationship between LE8 and its subscale scores and serum klotho levels by restricted cubic spline fitting. (A) LE8 score and klotho; (B) health behaviors score and klotho; (C) Health factors score and klotho. Adjusted for age, sex, race, education, marital status, PIR, insurance status, alcohol consumption status, HCVD, and CKD. LE8, Life’s Essential 8.

Table 2presents the weighted linear regression analysis results, illustrating the correlation between LE8 score and serum klotho levels. In the fully adjusted Model 3, each one-point increase in LE8 score was associated with an approximate 1.32 pg/mL increase in serum klothol levels (β: 1.32; 95% CI 0.73, 1.91). Subjects with moderate and high CVH had higher levels of serum klotho compared to those with low CVH. The β (95% CI) were 21.88 (0.03, 43.73) and 59.88 (28.11, 91.65), respectively. Tests for trend revealed a significant and substantial rise in serum klotho levels among individuals grouped by CVH (P for trend < 0.001). At the same time, the results from the multivariate adjusted RCS analysis revealed a significant non-linear relationship between LE8 score and serum klotho levels (P for nonlinearity = 0.033; Fig. 2A).

**Table 2:** Weighted linear regression coefficients (β) and 95% confidence intervals for the association between LE8 and its subscale scores and serum klotho: The United States, 2007 to 2016.

	Model 1		Model 2		Model 3	
	β (95%CI)	P value	β (95%CI)	P value	β (95%CI)	P value
LE8 score						
Continuous	1.57(1.03, 2.11)	<0.0001	1.59(1.03, 2.15)	<0.0001	1.32(0.73, 1.91)	<0.0001
Low (0–49)	Reference		Reference		Reference	
Moderate (50–79)	23.84(3.67, 44.01)	0.021	27.21(6.53, 47.90)	0.011	21.88(0.03, 43.73)	0.0497
High (80–100)	72.43(42.83, 102.03)	<0.0001	72.62(41.77, 103.48)	<0.0001	59.88(28.11, 91.65)	<0.001
P for trend		<0.0001		<0.0001		<0.001
Health behaviors score						
Continuous	0.65(0.26, 1.04)	0.001	0.76(0.36, 1.16)	<0.001	0.48(0.07, 0.88)	0.022
Low (0–49)	Reference		Reference		Reference	
Moderate (50–79)	16.57(−0.75, 33.89)	0.061	19.88(2.66, 37.09)	0.024	12.1(−5.72, 29.93)	0.179
High (80–100)	40.04(18.62, 61.45)	<0.001	44.92(22.71, 67.13)	<0.001	30.36(7.98, 52.73)	0.009
P for trend		<0.001		<0.001		0.007
Health factors score						
Continuous	1.27(0.74, 1.79)	<0.0001	1.16(0.66, 1.67)	<0.0001	1.05(0.54, 1.56)	<0.001
Low (0–49)	Reference		Reference		Reference	
Moderate (50–79)	0.65(−15.62, 16.91)	0.937	3.04(−13.03, 19.10)	0.708	−3.03(−20.09, 14.04)	0.724
High (80–100)	56.82(29.52, 84.12)	<0.0001	51.52(25.31, 77.73)	<0.001	43.59(17.37, 69.82)	0.002
P for trend		<0.0001		<0.001		<0.001

Abbreviation: LE8, Life’s Essential 8; CI, Confidence interval.

Model 1: Unadjusted.

Model 2: Adjusted for age, sex, and race.

Model 3: Adjusted for age, sex, race, education, marital status, PIR, insurance status, alcohol consumption status, HCVD, and CKD.

### 3.3 Association between health behaviors score and health factors score and serum klotho

Our data revealed a positive correlation between the health behaviors score and health factors score with serum klotho levels in the fully adjusted Model 3 (Table 2). When the aforementioned health behaviors score and health factors score were incorporated as continuous variables in the weighted linear regression model, their β (95% CI) with serum klotho levels were 0.48 (0.07, 0.88) and 1.05 (0.54, 1.56), respectively. The above results suggest that health factor score, which are derived from a combination of BMI, blood pressure, blood glucose, and blood lipids, may exhibit a stronger correlation with serum klotho levels. Upon conducting a more detailed analysis of the different groupings, it was found that significant associations were only observed between serum klotho and the groups characterized by high health behaviors and health factors, when compared to the reference group with low health behaviors and health factors. Furthermore, the dose-response relationship revealed a linear correlation between the health behaviors score and serum klotho levels (P for nonlinearity = 0.767; Fig. 2B), whereas the correlation between the health factors score and serum klotho levels was found to be non-linear (P for nonlinearity < 0.001; Fig. 2C).

### 3.4 Association between components of LE8 score and serum klotho

In **Supplementary Table 4**, the fully adjusted weighted linear regression models revealed significant positive associations between tobacco exposure score, BMI score, blood lipids score, and blood pressure score of LE8 components with serum klotho levels.The β (95% CI) for serum klotho was 0.36 (0.09, 0.62) for each 1-point increase in tobacco exposure score. The results of the group analysis and trend test for tobacco exposure in subsequent analyses showed a significant correlation (P < 0.05). Additionally, Analysis of BMI score (β: 0.43; 95% CI 0.18, 0.69), blood lipids score (β: 0.66; 95% CI 0.37, 0.95), and blood pressure score (β: 0.27; 95% CI 0.01−0.54) as continuous variables revealed a positive correlation with elevated levels of serum klotho. However, the association disappeared when blood pressure score were grouped and tested for trend. In addition, compared to the low-diet healthy group, the high-diet healthy group showed an increase in serum klotho levels (β: 25.17; 95% CI 3.67, 46.68), with a noticeable trend of increase (P for trend = 0.022).

### 3.5 Subgroup and sensitivity analyses

**Supplementary Table 5** presents the findings from the subgroup analysis, which examined the impact of age, sex, race, and CKD on the results of this study. For the LE8 score, we discovered a significant interaction between sex and race (P for interaction <0.001 and 0.009, respectively). The positive correlation between LE8 score and serum klotho was found to be even more significant in female and non-Hispanic black participants. Furthermore, the subgroup analysis of age and CKD did not reveal any significant interaction, indicating that the results remained consistent in this particular population (all P for interaction > 0.05). Simultaneously, health behavior score displayed interactions with different race subgroups (P for interaction = 0.009), while health factor score exhibited interactions with various sex subgroups (P for interaction <0.001). This finding could potentially provide an explanation for the observed interaction between the LE8 score and both sex and race subgroups.

Overall, the sensitivity analysis, which excluded participants with missing covariates and CVD, yielded consistent results with the main findings of this study. This suggests that the sensitivity analysis results were robust and reliable **(Supplementary Tables 6 and 7)**.

## 4 Discussion

This study is the first to uncover the correlation between CVH as measured by the LE8 score and the anti-aging marker klotho in a representative US middle-aged and older population. Notably, our findings demonstrate that serum klotho levels rise with higher LE8 score. At the same time, we also discovered that this correlation was evident in health behaviors score and health factors score. In a subgroup analysis, we found that the correlation between LE8 score and serum klotho was particularly strong among female and non-Hispanic black participants. These findings underscore the potential advantages of maintaining optimal CVH levels in terms of delaying the aging process.

The introduction of the LE8 score can effectively quantify the concept of CVH and allow individuals to easily self-assess their own CVH, which holds great importance in healthcare. Recent studies have provided strong evidence that maintaining a higher CVH level can greatly reduce the risk of all-cause and cardiovascular mortality [19]. These findings emphasize the importance of prioritizing primary care and prevention efforts to effectively reduce the likelihood of premature death in older age. In the meantime, LE8 indicators were found to be strongly correlated with a significant increase in life expectancy. By maintaining ideal CVH, individuals can expect to live 8.9 years longer at the age of 50. Furthermore, this increase in life expectancy can be attributed to a remarkable 40% reduction in cardiovascular-related deaths [4]. In summary, the LE8 score, which measures ideal CVH, is a powerful tool that can effectively reduce the public health burden of CVD and improve an individual's quality of life.

Significantly, maintaining optimal CVH may have advantageous effects in slowing down the aging process [2]. This supports our discovery that high scores on LE8 are linked to increased levels of the anti-aging marker klotho. Klotho is widely acknowledged as a vital protein in the fight against aging, as it plays a significant role in regulating oxidative stress and signaling pathways that are linked to the aging process [6]. Recent studies have revealed a significant link between LE8 score and accelerated aging, with oxidative stress biomarkers playing a crucial regulatory role [20]. As a result, our findings hold significant implications for gaining a deeper understanding of the intricate connection between CVH, oxidative stress, and the aging process. It is worth mentioning that our study found no association between physical activity score and serum klotho concentration. The link between physical activity and oxidative stress is complex, influenced by its type and intensity [21]. The NHANES physical activity questionnaire’s lack of detail may confound the true nature of this relationship. Additionally, physical activity may influence oxidative stress biomarkers independently of klotho pathways. Further research is necessary to unravel these intricate connections.

Klotho, named after the fate guardian god in Greek mythology, was initially discovered in a knockout mouse model that exhibits aging characteristics similar to those observed in humans [22]. There is research indicates that klotho has the potential to promote CVH by regulating endothelial function, reducing inflammation, and mitigating oxidative stress [23]. A recent cohort study has demonstrated a noteworthy association between elevated klotho levels and a substantial decrease in the risk of new CVD and all-cause mortality among hemodialysis patients [24]. In addition, recent NHANES-based studies have discovered that older adults with lower serum klotho levels are more likely to have risk factors for CVD [9]. These risk factors include higher BMI, smoking, alcohol consumption, and abnormal lipid levels [9]. Our findings revealed a notable elevation of approximately 60 pg/mL in klotho levels among individuals with high CVH, with reference to those with low CVH. However, it remains uncertain whether this observed effect is intensified under diseased states or in the presence of other conditions influencing klotho levels. Interestingly, research indicates that klotho serves as a valuable indicator for assessing the influence of lifestyle modifications on individual health outcomes [25]. Further investigation is warranted to determine the potential of klotho as an additional marker for monitoring cardiovascular health. Collectively, results from these studies collectively suggest a robust correlation between CVH and klotho levels, indicating klotho’s potential role as a connecting link between CVH and the aging process.

Notably, the findings from the subgroup analysis revealed a interaction between the positive impact of LE8 score on serum klotho levels and sex and race, with more pronounced associations observed in female and individuals of non-Hispanic black. These findings indicate that LE8 represents an enhancement in quantifying CVH methods, enabling it to more accurately detect variations between individuals and groups. Differences between sexes may be attributed to variances in biological structure and lifestyle factors [26]. Results from a national longitudinal study indicate that female tend to exhibit healthier lifestyles compared to male [27]. At the same time, klotho is regulated by estrogen, which emphasizes the existence of sex differences [28]. This aligns with our findings that sex differences were observed only in health factors in subsequent analyses, rather than in health behaviors. However, the current study lacks relevant research on the relationship between CVH and race, making it difficult to provide a more detailed explanation of ethnic differences in subgroup analysis. Further studies are needed to address this gap and improve our understanding in the future. Our current findings can only suggest one direction, and it is possible that this difference may exist in various health behaviors.

Our study possesses several notable strengths. First and foremost, our data is derived from a nationally representative sample of individuals, allowing for better generalizability to the overall population. Furthermore, we have established a dose-response relationship between CVH and klotho, and taking into account potential confounding factors. Additionally, our subgroup analyses have revealed variations in CVH based on gender and ethnicity. However, it is important to take into account the limitations. The cross-sectional nature of our study design limits our ability to establish causal relationships. Additionally, our capacity to dynamically monitor cardiovascular health and changes in serum klotho is significantly restricted. The use of self-reported questionnaires introduces the potential for recall bias. Furthermore, despite adjusting for potential confounders, residual confounding may persist.

## 5 Conclusion

In this study, which included a representative sample of middle-aged and older in the United States, we found a strong and positive correlation between LE8 score, health behavior score, and health factor score with klotho levels. Interestingly, health factors had a significantly stronger association with klotho levels compared to health behaviors. Specifically, factors such as BMI, lipid profile, and blood pressure had more significant effects on health factors. The results from the RCS analysis suggest a non-linear association between LE8 score and health factors score and klotho levels. On the other hand, the relationship between health behaviors score and klotho levels appears to be linear. Furthermore, the findings from the subgroup analysis revealed a strong correlation between LE8 score and serum klotho levels, particularly among female and non-hispanic black participants. The findings suggest that maintaining optimal CVH could potentially enhance the anti-aging marker klotho levels and prevent aging. However, further studies with stronger evidence are required to establish a cause-and-effect relationship.

## Contributors

KK, PF-L and DD contributed to the conception and design, acquisition, analysis, interpretation of the data, and drafting of the manuscript or critical revision for important intellectual content. XL-P, AA and DK completed the software analysis and data visualization. JD-L, XZ-L, MA and QW collected and organized data. XM and YT-M contributed to the conception and design and reviewing of the manuscript or critical revision for important intellectual content. All authors approved the final version, and agree to be accountable for all aspects of the work.

## Funding

This study was supported by the Key Research and Development Task Special in Xinjiang Uygur Autonomous Region (Grant No. 2022B03022-3) and national natural science foundation of China (Grant No. 82360090).

## Availability of data and materials

This study is a secondary analysis based on a publicly available database and the raw data can be found on the website: https://www.cdc.gov/nchs/nhanes/index.htm.

## Declarations

### Competing interests

None declared.

### Consent for publication

No applicable.

### Ethics declarations

The study protocols of NHANES were approved by the NCHS Research Ethics Review Board and participant written informed consent was obtained (https://www.cdc.gov/nchs/nhanes/irba98.htm). The additional ethical review was no longer required for the present study due to the usage of publicly available data without identifiable personal information.
