# Supplementary material for: Maintaining ideal cardiovascular health is associated with higher serum anti-aging protein klotho in the middle-aged and older populations
Source: J Nutr Health Aging. 2024 Apr 5;28(6):100224. doi: 10.1016/j.jnha.2024.100224 (PMC12275714; doi:10.1016/j.jnha.2024.100224)
Supplement: Supplementary file 1 [file mmc1.docx]

Supplementary Material

**Maintaining ideal cardiovascular health is associated with higher serum anti-aging protein klotho in the middle-aged and older populations**

Kaisaierjiang Kadier^1^*, Pengfei Liu^1^*, Diliyaer Dilixiati^2^*, Xinliang Peng^1^, Aikeliyaer Ainiwaer^3^, Dinigeer Kadier^4^, Jiande Lu^5^, Xiaozhu Liu^6^, Mierxiati Ainiwan^1^, Qi Wang^1^, Xiang Ma^1^, and Yitong Ma^1^

1. Department of Cardiology, First Affiliated Hospital of Xinjiang Medical University, Urumqi, China

2. Department of Urology, First Affiliated Hospital of Xinjiang Medical University, Urumqi, China

3. Department of Physiology, Cardiovascular Research Institute Maastricht (CARIM), Maastricht, Netherlands

4. Data Science and Business Analytics, Faculty of Economics and Business, University of Amsterdam, Amsterdam, Netherlands

1. Department of General Surgery, Children's Hospital of Xinjiang Uygur Autonomous Region, Urumqi, China
2. Department of Critical Care Medicine, Beijing Shijitan Hospital, Capital Medical University, Beijing, China

**Equal contributions:** ^*^These authors have contributed equally to this work and share first authorship

**Supplementary Table 1** Characteristics of participants excluded from this study due to missing data (n=8,563)

**Supplementary Table 2** Measurement and Scoring of Life’s Essential 8

**Supplementary Table 3** The numbers and percentages of missing covariate data

**Supplementary Table 4** Weighted linear regression coefficients (β) and 95% confidence intervals for the association between components of LE8 score and serum klotho: The United States, 2007 to 2016

**Supplementary Table 5** Subgroup analysis for the association between LE8 and its subscale scores and serum klotho.

**Supplementary Table 6** Weighted linear regression coefficients (β) and 95% confidence intervals for the association between LE8 and its subscale scores and serum klotho: The United States, 2007 to 2016 (exclude participants with missing covariates)

**Supplementary Table 7** Weighted linear regression coefficients (β) and 95% confidence intervals for the association between LE8 and its subscale scores and serum klotho: The United States, 2007 to 2016 (exclude participants with cardiovascular disease)

**Supplementary Table 1** Characteristics of participants excluded from this study due to missing data (n=8,563)

| Characters | Overall  (n=8,563) |
| --- | --- |
| **Age,year** | 60.14±0.24 |
| **Sex** |  |
| Male | 4216(48.01) |
| Female | 4347(51.99) |
| **Race** |  |
| Mexican American | 1161( 7.15) |
| Non-Hispanic Black | 2042(13.94) |
| Non-Hispanic White | 3390(63.99) |
| Others | 1970(14.92) |

Continuous variables are presented as the weighted mean ± standard deviation, and categorical variables are presented as n (weighted percentages).

As only demographic basic characteristics were considered, the use of household interview weights was restricted to weighting purposes.

**Supplementary Table 2** Measurement and Scoring of Life’s Essential 8

| Domain | CVH Metric | Measurement | Quantification and Scoring of CVH Metric |
| --- | --- | --- | --- |
| Health behaviors | Diet | Healthy Eating Index-2015 diet score percentile | Quantiles of HEI-2015 (population)  **Scoring (Population):**  Points Quantile  100 ≥95th percentile (top/ideal diet)  80 75th – 94th percentile  50 50th – 74th percentile  25 25th – 49th percentile  0 1st – 24th percentile (bottom/least ideal quartile) |
|  | Physical activity | Self-reported minutes of moderate or vigorous physical activity per week | **Metric:** Minutes of moderate (or greater) intensity activity per week  **Scoring:**  Points Minutes  100 ≥150  90 120 – 149  80 90 – 119  60 60 – 89  40 30 – 59  20 1 – 29  0 0 |
|  | Nicotine exposure | Self-reported use of cigarettes or inhaled nicotine-delivery system | **Metric:** Combustible tobacco use and/or inhaled NDS use; or secondhand smoke exposure  **Scoring:**  Points Status  100 Never smoker  75 Former smoker, quit ≥5 y  50 Former smoker, quit 1 - <5 y  25 Former smoker, quit <1 year, or currently using inhaled NDS  0 Current smoker  Subtract 20 points (unless score is 0) for living with active indoor smoker in home |
|  | Sleep health | Self-reported average hours of sleep per night | **Metric:** Average hours of sleep per night  **Scoring:**  Points Level  100 7 – <9  90 9 – <10  70 6 – <7  40 5 – <6 or ≥10  20 4 – <5  0 <4 |
| Health factors | Body mass index | Body weight (kg) divided by height squared (m^2^) | **Metric:** Body mass index (kg/m^2^)  **Scoring:**  Points Level  100 <25  70 25.0 – 29.9  30 30.0 – 34.9  15 35.0 – 39.9  0 ≥40.0 |
|  | Blood lipids | Plasma total and HDL cholesterol with calculation of non-HDL cholesterol | **Metric:** Non-HDL-cholesterol (mg/dL)  **Scoring:**  Points Level  100 <130  60 130 – 159  40 160 – 189  20 190 – 219  0 ≥220  If drug-treated level, subtract 20 points |
|  | Blood glucose | Fasting blood glucose or casual hemoglobin A1c | **Metric:** Fasting blood glucose (mg/dL) or Hemoglobin A1c (%)  **Scoring:**  Points Level  100 No history of diabetes and FBG <100 (or HbA1c < 5.7)  60 No diabetes and FBG 100 – 125 (or HbA1c 5.7-6.4) (Pre-diabetes)  40 Diabetes with HbA1c <7.0  30 Diabetes with HbA1c 7.0 – 7.9  20 Diabetes with HbA1c 8.0 – 8.9  10 Diabetes with Hb A1c 9.0 – 9.9  0 Diabetes with HbA1c ≥10.0 |
|  | Blood pressure | Appropriately measured systolic and diastolic blood pressure | **Metric:** Systolic and diastolic blood pressure (mm Hg)  **Scoring:**  Points Level  100 <120/<80 (Optimal)  75 120-129/<80 (Elevated)  50 130-139 or 80-89 (Stage I HTN)  25 140-159 or 90-99  0 ≥160 or ≥100  Subtract 20 points if treated level |

**Supplementary Table 3** The numbers and percentages of missing covariate data

| **Covariate** | **Numbers** | **Percentages (%)** |
| --- | --- | --- |
| Cardiovascular disease | 2 | 0.02 |
| Insurance | 4 | 0.04 |
| Marital | 4 | 0.04 |
| Education | 6 | 0.06 |
| Chronic kidney disease | 41 | 0.43 |
| HCVD | 231 | 2.44 |
| Alcohol consumption status | 382 | 4.04 |
| Poverty-income ratio | 723 | 7.65 |

**Supplementary Table 4** Weighted linear regression coefficients (β) and 95% confidence intervals for the association between components of LE8 score and serum klotho: The United States, 2007 to 2016

|  | Model 1 | |  | Model 2 | |  | Model 3 | |
| --- | --- | --- | --- | --- | --- | --- | --- | --- |
|  | β (95%CI) | P value |  | β (95%CI) | P value |  | β (95%CI) | P value |
| **HEI-2015 diet score** |  |  |  |  |  |  |  |  |
| Continuous | 0.39(0.09, 0.69) | 0.011 |  | 0.4(0.10-0.70) | 0.009 |  | 0.28(-0.01, 0.57) | 0.057 |
| Low (0–49) | Reference |  |  | Reference |  |  | Reference |  |
| Moderate (50–79) | 12.17(-9.22, 33.56) | 0.261 |  | 14.28(-7.15, 35.70) | 0.188 |  | 11.29(-8.97, 31.55) | 0.270 |
| High (80–100) | 33.32(11.71, 54.92) | 0.003 |  | 34.02(12.24, 55.81) | 0.003 |  | 25.17(3.67, 46.68) | 0.023 |
| P for trend |  | 0.003 |  |  | 0.003 |  |  | 0.022 |
| **Physical activity score** |  |  |  |  |  |  |  |  |
| Continuous | 0.03(-0.18, 0.23) | 0.792 |  | 0.06(-0.16, 0.28) | 0.581 |  | 0.03(-0.20, 0.25) | 0.813 |
| Low (0–49) | Reference |  |  | Reference |  |  | Reference |  |
| Moderate (50–79) | -15.29(-56.95, 26.37) | 0.467 |  | -18.05(-60.52, 24.41) | 0.400 |  | -21.7(-63.58, 20.17) | 0.304 |
| High (80–100) | 4.26(-15.20, 23.72) | 0.664 |  | 6.97(-13.68, 27.61) | 0.503 |  | 3.59(-17.32, 24.50) | 0.733 |
| P for trend |  | 0.630 |  |  | 0.467 |  |  | 0.685 |
| **Tobacco exposure score** | |  |  |  |  |  |  |  |
| Continuous | 0.53(0.28, 0.77) | <0.0001 |  | 0.53(0.29, 0.78) | <0.0001 |  | 0.36(0.09, 0.62) | 0.009 |
| Low (0–49) | Reference |  |  | Reference |  |  | Reference |  |
| Moderate (50–79) | 19.93(-5.72, 45.58) | 0.126 |  | 34.4(9.89, 58.91) | 0.007 |  | 25.36(1.32, 49.40) | 0.039 |
| High (80–100) | 51.87(28.16, 75.58) | <0.0001 |  | 51.66(28.01, 75.32) | <0.0001 |  | 34.28(8.38, 60.19) | 0.010 |
| P for trend |  | <0.0001 |  |  | <0.0001 |  |  | 0.010 |
| **Sleep health score** |  |  |  |  |  |  |  |  |
| Continuous | -0.25(-0.56, 0.06) | 0.107 |  | -0.07(-0.39, 0.24) | 0.640 |  | -0.16(-0.49, 0.18) | 0.357 |
| Low (0–49) | Reference |  |  | Reference |  |  | Reference |  |
| Moderate (50–79) | 10.14(-12.27, 32.54) | 0.370 |  | 18.36(-3.73, 40.46) | 0.102 |  | 13.79(-8.66, 36.25) | 0.224 |
| High (80–100) | -14.53(-36.48, 7.42) | 0.191 |  | -2.28(-24.81, 20.26) | 0.841 |  | -7.42(-31.35, 16.51) | 0.537 |
| P for trend |  | 0.046 |  |  | 0.354 |  |  | 0.202 |
| **Body mass index score** |  |  |  |  |  |  |  |  |
| Continuous | 0.42(0.16, 0.67) | 0.002 |  | 0.44(0.19, 0.70) | <0.001 |  | 0.43(0.18, 0.69) | 0.001 |
| Low (0–49) | Reference |  |  | 1 (Reference) |  |  | Reference |  |
| Moderate (50–79) | 4.95(-13.00, 22.91) | 0.584 |  | 11.55(-6.37, 29.46) | 0.203 |  | 10.29(-8.23, 28.81) | 0.271 |
| High (80–100) | 40.33(17.71, 62.95) | <0.001 |  | 38.21(15.50, 60.92) | 0.001 |  | 37.46(14.94, 59.97) | 0.001 |
| P for trend |  | 0.002 |  |  | 0.002 |  |  | 0.002 |
| **Blood lipids score** | |  |  |  |  |  |  |  |
| Continuous | 0.77(0.48, 1.05) | <0.0001 |  | 0.7(0.42, 0.99) | <0.0001 |  | 0.66(0.37, 0.95) | <0.0001 |
| Low (0–49) | Reference |  |  | Reference |  |  | Reference |  |
| Moderate (50–79) | 56.11(34.70, 77.51) | <0.0001 |  | 50.63(29.19, 72.06) | <0.0001 |  | 48.42(26.69, 70.15) | <0.0001 |
| High (80–100) | 37.69(18.88, 56.50) | <0.001 |  | 37.05(18.12, 55.98) | <0.001 |  | 34.82(15.84, 53.79) | <0.001 |
| P for trend |  | 0.001 |  |  | 0.001 |  |  | 0.002 |
| **Blood glucose score** |  |  |  |  |  |  |  |  |
| Continuous | 0.12(-0.17, 0.41) | 0.426 |  | 0.05(-0.23, 0.34) | 0.707 |  | -0.03(-0.31, 0.25) | 0.814 |
| Low (0–49) | Reference |  |  | Reference |  |  | Reference |  |
| Moderate (50–79) | -5.56(-27.90, 16.78) | 0.622 |  | -8.68(-31.21, 13.86) | 0.445 |  | -11.45(-34.76, 11.86) | 0.330 |
| High (80–100) | 14.5(-8.61, 37.61) | 0.215 |  | 9.78(-12.92, 32.49) | 0.393 |  | 4.5(-18.02, 27.01) | 0.691 |
| P for trend |  | 0.045 |  |  | 0.100 |  |  | 0.219 |
| **Blood pressure score** |  |  |  |  |  |  |  |  |
| Continuous | 0.49(0.23, 0.75) | <0.001 |  | 0.38(0.12, 0.65) | 0.005 |  | 0.27(0.01, 0.54) | 0.041 |
| Low (0–49) | Reference |  |  | Reference |  |  | Reference |  |
| Moderate (50–79) | -1.91(-21.90, 18.08) | 0.849 |  | -4.17(-24.70, 16.37) | 0.687 |  | -10.57(-30.98, 9.83) | 0.304 |
| High (80–100) | 28.38(6.09, 50.67) | 0.013 |  | 21.54(-1.18, 44.27) | 0.063 |  | 13.53(-8.80, 35.86) | 0.230 |
| P for trend |  | 0.003 |  |  | 0.018 |  |  | 0.072 |

Abbreviation: LE8, Life’s Essential 8; HEI-2015, 2015 Healthy Eating Index; CI, Confidence interval

Model 1: Unadjusted

Model 2: Adjusted for age, sex, and race

Model 3: Adjusted for age, sex, race, education, marital status, PIR, insurance status, alcohol consumption status, HCVD, and CKD

**Supplementary Table 5** Subgroup analysis for the association between LE8 and its subscale scores and serum klotho.

|  | **LE8 score** | | |  | **Health behaviors score** | | |  | **Health factors score** | | |
| --- | --- | --- | --- | --- | --- | --- | --- | --- | --- | --- | --- |
|  | β (95%CI) | p value | P for interaction |  | β (95%CI) | p value | P for interaction |  | β (95%CI) | p value | P for interaction |
| **Age** |  |  | 0.554 |  |  |  | 0.630 |  |  |  | 0.206 |
| 40-59y | 1.5( 0.78, 2.21) | <0.0001 |  |  | 0.45( -0.04, 0.93) | 0.069 |  |  | 1.29( 0.65, 1.93) | <0.001 |  |
| 60-79y | 1.08( 0.23, 1.92) | 0.013 |  |  | 0.65( 0.06, 1.23) | 0.032 |  |  | 0.56( -0.17, 1.29) | 0.130 |  |
| **Sex** |  |  | <0.001 |  |  |  | 0.138 |  |  |  | <0.0001 |
| Male | 0.14( -0.71, 1.00) | 0.741 |  |  | 0.27( -0.39, 0.92) | 0.417 |  |  | -0.15( -0.86, 0.55) | 0.665 |  |
| Female | 2.2( 1.36, 3.04) | <0.0001 |  |  | 0.61( 0.12, 1.11) | 0.016 |  |  | 1.99( 1.23, 2.74) | <0.0001 |  |
| **Race** |  |  | 0.009 |  |  |  | 0.009 |  |  |  | 0.251 |
| Non-Hispanic white | 1.2( 0.46, 1.94) | 0.002 |  |  | 0.33( -0.22, 0.88) | 0.237 |  |  | 1.1( 0.47, 1.73) | <0.001 |  |
| Non-Hispanic black | 2.6( 1.19, 4.02) | <0.001 |  |  | 1.04( 0.01, 2.08) | 0.049 |  |  | 1.68( 0.55, 2.81) | 0.004 |  |
| Mexican American | -0.05( -1.39, 1.28) | 0.934 |  |  | -0.23( -1.07, 0.62) | 0.591 |  |  | 0.22( -0.67, 1.10) | 0.622 |  |
| Other | 2.15( 0.48, 3.83) | 0.013 |  |  | 1.4( 0.39, 2.41) | 0.007 |  |  | 1.04( -0.26, 2.35) | 0.114 |  |
| **CKD** |  |  | 0.530 |  |  |  | 0.203 |  |  |  | 0.799 |
| Yes | 1.57( 0.09, 3.05) | 0.037 |  |  | 1.04( 0.04, 2.05) | 0.041 |  |  | 1.12( 0.60, 1.65) | <0.0001 |  |
| No | 1.28(0.68, 1.88) | <0.0001 |  |  | 0.37( -0.03, 0.77) | 0.067 |  |  | 0.78( -0.46, 2.01) | 0.212 |  |

Abbreviation: LE8, Life’s Essential 8; CI, Confidence interval; CKD, Chronic kidney disease

Adjusted for age, sex, race, education, marital status, PIR, insurance status, alcohol consumption status, HCVD, and CKD when they were not the strata variables

**Supplementary Table 6** Weighted linear regression coefficients (β) and 95% confidence intervals for the association between LE8 and its subscale scores and serum klotho: The United States, 2007 to 2016 (exclude participants with missing covariates)

|  | | **Fully adjusted model** | |
| --- | --- | --- | --- |
|  | | β (95%CI) | P value |
| **LE8 score** | |  |  |
| Continuous | | 1.23( 0.62, 1.84) | <0.001 |
| Low (0–49) | | Reference |  |
| Moderate (50–79) | | 18.89( -3.68, 41.46) | 0.099 |
| High (80–100) | | 52.98( 20.46, 85.49) | 0.002 |
| P for trend | |  | 0.002 |
|  |  |  |  |
| **Health behaviors score** | |  |  |
| Continuous | | 0.44( 0.01, 0.86) | 0.043 |
| Low (0–49) | | Reference |  |
| Moderate (50–79) | | 9.89( -9.38, 29.16) | 0.309 |
| High (80–100) | | 29.23( 5.34, 53.11) | 0.017 |
| P for trend | |  | 0.014 |
|  | |  |  |
| **Health factors score** | |  |  |
| Continuous | | 0.99( 0.48, 1.50) | <0.001 |
| Low (0–49) | | Reference |  |
| Moderate (50–79) | | -7.79(-26.43, 10.85) | 0.407 |
| High (80–100) | | 40.31( 14.34, 66.28) | 0.003 |
| P for trend | |  | 0.001 |

Abbreviation: LE8, Life’s Essential 8; CI, Confidence interval

Adjusted for age, sex, race, education, marital status, PIR, insurance status, alcohol consumption status, HCVD, and C

**Supplementary Table 7** Weighted linear regression coefficients (β) and 95% confidence intervals for the association between LE8 and its subscale scores and serum klotho: The United States, 2007 to 2016 (exclude participants with cardiovascular disease)

|  | | **Fully adjusted model** | |
| --- | --- | --- | --- |
|  | | β (95%CI) | P value |
| **LE8 score** | |  |  |
| Continuous | | 1.35( 0.73, 1.97) | <0.0001 |
| Low (0–49) | | Reference |  |
| Moderate (50–79) | | 26.7( 4.86, 48.54) | 0.017 |
| High (80–100) | | 63.53( 31.06, 95.99) | <0.001 |
| P for trend | |  | <0.001 |
|  |  |  |  |
| **Health behaviors score** | |  |  |
| Continuous | | 0.46( 0.05, 0.88) | 0.029 |
| Low (0–49) | | Reference |  |
| Moderate (50–79) | | 13.58( -5.66, 32.81) | 0.163 |
| High (80–100) | | 31.11( 7.47, 54.75) | 0.011 |
| P for trend | |  | 0.009 |
|  | |  |  |
| **Health factors score** | |  |  |
| Continuous | | 1.09( 0.54, 1.64) | <0.001 |
| Low (0–49) | | Reference |  |
| Moderate (50–79) | | -0.33(-19.87, 19.21) | 0.973 |
| High (80–100) | | 46.68( 19.36, 74.00) | 0.001 |
| P for trend | |  | <0.001 |

Abbreviation: LE8, Life’s Essential 8; CI, Confidence interval

Adjusted for age, sex, race, education, marital status, PIR, insurance status, alcohol consumption status, HCVD, and CKD
